# Prevalence of occupational exposure to needle‐stick injury and associated factors among healthcare workers of developing countries: Systematic review

**DOI:** 10.1002/1348-9585.12179

**Published:** 2020-12-12

**Authors:** Dechasa Adare Mengistu, Sina Temesgen Tolera

**Affiliations:** ^1^ Department of Environmental Health Science College of Health and Medical Science Haramaya University Harar Ethiopia

**Keywords:** developing countries, healthcare system, healthcare workers, needle‐stick injury, occupational exposure

## Abstract

**Background:**

Healthcare workers are at high risk of infection from blood‐borne pathogens, such as Hepatitis B and C Virus, and Human Immunodeficiency. Occupational exposure to needle‐stick injuries (NSIs) continue to have a major health problem in the healthcare systems of developing countries. Thus, this review article aimed to provide the evidence on the prevalence of NSI and associated factors among healthcare workers of developing countries.

**Methods:**

The studies published from 2012 to 2019 were identified through systematic searches of electronic databases such as PubMed, Google Scholar, CINAHL, MEDLINE, Scopus, Med Nar, and Science Direct. The MeSH terms and/or keywords was used in conjunction with “AND” or “OR” (Boolean logic operators). All identified keywords and an index terms were checked across the included databases. Assessment and evaluation were taken to confirm the quality and relevance of the included articles, followed by extraction and analysis of data.

**Result:**

Overall, 2021 articles were identified using specified search terms from the initial searches of the literature (2012‐2019). A total of 13 articles met eligibility criteria were included in the review. Among 6513 participants, 1009 and 2201 participants involved to determine 1‐year and throughout career prevalence, respectively. The prevalence of NSI ranged from 19.9% to 54.0% with an overall prevalence of 35.7% and 38.5 to 100% with an overall prevalence of 64.1% in the previous 1 year and throughout career, respectively. Sex, workload, needle recapping, overuse of injection, and practice of universal precautions, training, occupation, working experience, and personal protective equipment were among the factors associated with the prevalence of NSIs in developing countries.

**Conclusion:**

The review indicated that NSIs have been identified as one of the most serious issues that affect the health and well‐being of healthcare workers in the majority of healthcare systems of developing countries. There is a need to apply safety practices or other measures to reduce the risk of NSIs.

## 1 INTRODUCTION

Needle‐stick injury (NSI) is defined as: “introduction into the body of health care providers during the routine performance of their duties of blood or other potentially hazardous materials by a hollow bore needle or sharp instruments eg needles, lancets, and contaminated broken glass”.^1^

Healthcare workers are at the highest risk of occupational infections from biological factors in their working environment, which is a major risk factor in the transmission of infection as they exposed to body fluids from day to day activities. Exposure to blood and body fluids and NSIs in an occupational setting affects the safety and wellbeing of healthcare workers and compromises the quality of health care delivered.^2^

Annually, hundreds of thousands of healthcare workers are at risk of work‐related diseases such as blood‐borne diseases as the result of needle sticks and sharp injuries.^3^, ^4^ Accidental injuries that include needle sticks and sharps injuries to healthcare workers continue to have a major problem in the healthcare systems that associated with various infections such as hepatitis B, hepatitis C, and human immunodeficiency viruses^5^, ^6^, ^7^, ^8^ and other blood‐borne pathogens such as cytomegalovirus, herpes simplex virus, and parvovirus B19.^9^

Worldwide, the incidence of occupational injury/disease from sharp objects including NSI among healthcare workers is estimated to be 3 million where a chance of four injuries per healthcare worker occurs every year^10^, ^11^ that accounts for 40% for hepatitis and 2.5% for HIV infection.^12^ Healthcare workers in developing countries, especially they are endemic in sub‐Saharan Africa, are at serious risk of infection from blood‐borne pathogens, particularly Hepatitis B Virus (HBV), Hepatitis C Virus (HCV), and Human Immunodeficiency Virus (HIV).^13^, ^14^

According to World Health Organization estimation, the annual exposure to blood pathogens through percutaneous accounts for three million worldwide, of which 2 million, 0.9 million, and 170 000 healthcare workers/professionals were exposed to hepatitis B Virus (70 000 contracted), Hepatitis C Virus (150 000 contracted) and Human Immunodeficiency Virus (500 contracted) respectively of which more than 90% occurred in developing countries especially sub‐Saharan Africa.^13^, ^14^

The centers for Disease Control and Prevention have developed standard precautions with a series of procedures for preventing occupational exposure and handling infectious materials such as blood and other body fluids that include regular personal hygiene; using protective barriers disposing of sharps and other clinical waste in an appropriate manner.^15^

The Occupational Safety and Health Administration (OSHA) blood‐borne pathogens standard also states that “safety including engineering and work practice controls shall be used to eliminate or minimize employee exposure”, such as the use of sharps with a safety‐engineered injury protection mechanism.^16^

Thus, this review article aimed to systematically review the previous evidence on the prevalence of NSI and associated factors among healthcare workers of developing countries.

## 2 METHODS

This systematic review was carried out in accordance with PRISMA (Preferred Reporting Items for Systematic Reviews and Meta‐Analysis) guidelines.^17^

### 2.1 Eligibility criteria

Articles with their full texts available in English with clear objective and methodology, studies included NSIs as a dependent variable and provided quantitative outcomes were included in this review. This review also considered studies that include healthcare workers involved in clinical practice at different departments of private and governmental health institutions of developing countries. Particularly, the review considered studies where the populations of interest were medical doctors, nurses/midwifes, auxiliary nurses, laboratory technicians, and medical students. The articles published from 2012 to 2019 were included in this review.

### 2.2 Information source and search strategy

The studies published from 2012 to 2019 were identified through systematic searches of electronic databases such as PubMed, Google Scholar, CINAHL, MEDLINE, Scopus, Med Nar, and Science Direct, followed by analysis of the text contained in the title and abstract. The keywords and MeSH terms were used individually or in conjunction with “AND” or “OR” (Boolean logic operators) as the following: (Prevalence∗OR frequency∗OR magnitude) AND (occupational*OR accidental occupational*OR work place) AND (exposure∗OR accident*OR hazard) AND (needle injury*OR needlestick injury*OR percutaneous injury) AND (associated∗OR risk∗OR related∗OR determinant) AND (factor∗OR factors) AND (healthcare worker*OR health worker∗OR medical personnel*OR health personnel∗OR health professional∗OR health care provider) AND (developing countries∗OR low income countries∗OR middle income countries).

Moreover manual searching for further studies was conducted by authors (Mengistu DA and Tolera ST) to cover other published articles not included in electronic databases. All identified keywords and an index term were checked by authors (Mengistu DA and Tolera ST) across all the included databases. Finally, searching of further articles was conducted to cover the area missed. The last search was done on December 31, 2019.

### 2.3 Study selection

All duplicated searches were removed using the ENDNOTE software version X5 (Thomson Reuters, USA). The authors (Mengistu DA and Tolera ST) independently screened the titles and the abstracts of all identified articles by applying the inclusion criteria. Disagreement made among the authors was solved by taking the mean score of the two reviewers (Mengistu DA and Tolera ST) after discussing the rationale on differences. Finally, the review included the articles conducted on the prevalence of NSIs or NSIs and associated factors among healthcare workers in both governmental and private healthcare systems of developing countries.

### 2.4 Data extraction and quality assessment

A predefined form was used to extract information from selected studies under the following headings: author; sample size; year; country of study; study design; primary outcome (prevalence of NSI), and possible confounding factors considered. And, articles were evaluated by the authors (Mengistu DA and Tolera ST) to confirm its relevance to the study and to confirm the quality of the work. Then all required data about the prevalence of NSIs and possible associated factors were extracted from the eligible articles.

In addition, for articles meet inclusion criteria, abstracts were read to further establish their relevance to the study. All selected articles were subjected to a rigorous, independent appraisal using standardized critical appraisal tools (JBI Critical Appraisal tools) to determine the quality of the articles. Then the score was taken across all studies and graded as high (85% and above score), moderate (60%‐85% score), and low (<60% score) quality. Furthermore, disagreement on what is to be extracted was solved by discussion.

## 3 RESULTS

### 3.1 Study selection

The articles conducted on the prevalence of NSIs and/or associated factors in developing countries and published from 2012 to 2019 were searched using electronic databases. Overall, about 2021 articles were identified using specified search terms from the initial searches of the literature. From 2021 articles searched, 605 articles were excluded due to duplication while 1177 articles were excluded during screening. Furthermore, of 239 full‐text articles assessed to determine their eligibility for including in the systematic review, 226 articles were excluded due to unclear objectives, unclear methodology, and not healthcare workers participants.

**FIGURE 1:**
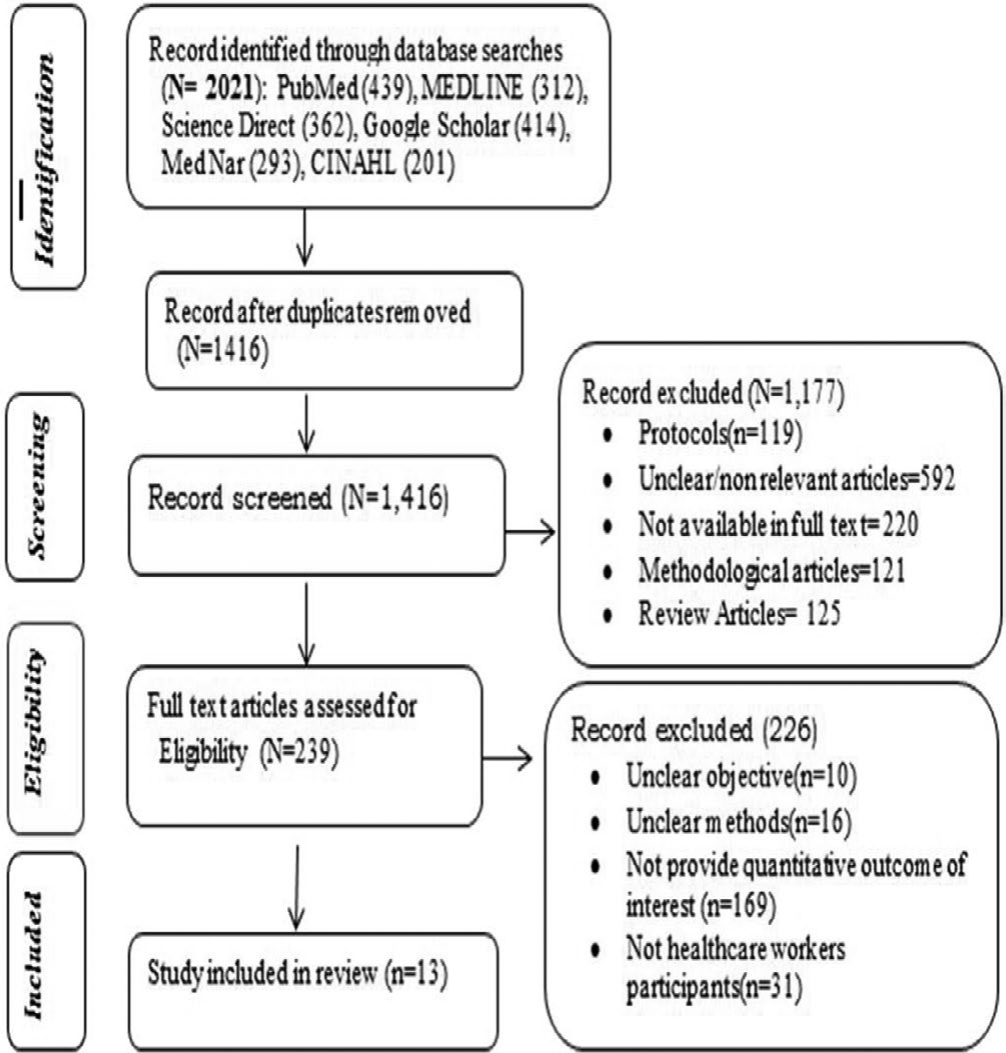
Schematic illustration of the patient enrollment procedure. PRISMA flowchart showing the process of study selection for reviewing the evidence on occupational exposure to needle stick injuries and associated factors among HCWs of developing countries (2012 ‐ 2019)

Finally, a total of 13 articles that assess the prevalence of NSIs and/or factors associated with NSIs were included. The selection process and reason for exclusion are stated in Figure 1 below.

### 3.2 Characteristics of the included studies

A total of 13 articles^18^, ^19^, ^20^, ^21^, ^22^, ^23^, ^24^, ^25^, ^26^, ^27^, ^28^, ^29^, ^30^ reported the prevalence of NSI or factors associated with the prevalence of NSI during the previous 1 year and/or throughout the career time were included in the systematic review yielding an overall 6513 healthcare workers ranged from 102^23^ to 2156^29^ study participants.

Articles included in this review were reported quantitative value such as prevalence of NSI and/or associated factors with statistical tests. Among the studies included for systematically determining the evidence on the prevalence of NSI and associated factors, all reviewed articles were used cross‐sectional study design. The included articles were conducted in Egypt,^29^ Ethiopia,^20^, ^21^, ^27^, ^30^ India,^24^ Iran,^19^ Jordan,^22^ Malaysia,^18^ Pakistan,^28^ and Tanzania.^25^

Among the total of 13 articles included in the systematic review, the majority, 10 (76.9%) of the articles reported both the prevalence of NSI and associated factors^19^, ^20^, ^21^, ^30^ while one article reported only the prevalence of NSIs^18^ and the other two (15.38%) studies reported factors associated with the prevalence of NSIs.^22^, ^29^ Six articles^18^, ^19^, ^23^, ^24^, ^27^, ^30^ reported both 1 year and throughout career time NSIs exposure prevalence, while the rest of the studies reported either 1 year or throughout career time prevalence.^20^, ^21^, ^25^, ^26^, ^28^

Overall, the main results of all included articles are summarized in Tables 1 and 2, along with the quantitative outcome (prevalence), study design, sample size, study location, and associated factors.

**TABLE 1:** Shows overall characteristics of included studies for a systematic review on the prevalence of needle‐stick injury among healthcare workers of developing countries

Author (year)	Study design	Sample size	Prevalence of NSIs		Study participant	Risk of bias	Location	Reference
			Within the previous 12 mo	During their carrier				
Min Swe et al, 2014	Cross‐sectional	316	19.9%	100%	Medical students	Low	Malaysia	18
Jahangiri et al, 2016	Cross‐sectional	168	54.0%	76.0%	Nurses	Low	Iran	19
Feleke, 2013	Cross‐sectional	631	—	66.6%	Health professionals	Low	Ethiopia	20
Girmaye et al, 2018	Cross‐sectional	244	28.3%	—	Health professionals	Low	Ethiopia	21
Amira, 2014	Cross‐sectional	102	24.5%	40.2%	Healthcare workers	Low	Nigeria	23
Archana et al, 2018	Cross‐sectional	950	35.3%	68.3%	Health care providers	Low	India	24
Chalya et al, 2015	Cross‐sectional	436	31.65%	—	Healthcare workers	Low	Tanzania	25
Isara et al, 2015	Cross‐sectional	122	51.0%	—	Healthcare workers	Low	Nigeria	26
Kebede & Gerensea, 2018	Cross‐sectional	313	34.5%	48.8%	Nurses	Low	Ethiopia	27
Khan et al, 2013	Cross‐sectional	497	—	64.0%	Healthcare workers	Low	Pakistan	28
Weldesamuel et al, 2019	Cross‐sectional	456	25.9%	38.5%	Healthcare workers	Low	Ethiopia	30

**TABLE 2:** Shows characteristics of included studies for reviewing the evidence on factors associated with needle‐stick injury in developing countries

Author (year)	Study design	Sample size	Factors associated with needle stick injuries	Location	Reference
Jahangiri et al, 2016	Cross‐sectional	168	Sex (AOR = 0.24 and CI (0.095‐0.612), work load (OR = 0.86 and CI (0.812‐0.925).	Iran	19
Feleke, 2013	Cross‐sectional	645	Work load (AOR = 15.58, 95% CI: 7.78 −31.13), working in private hospitals (AOR = 3.03, 95% CI: 1.73‐5.31), disassembling of syringe and needle (AOR = 5.38, 95% CI: 2.68‐10.76), over use of injection (AOR = 5.65, 95% CI: 2.4‐13.3), universal precaution (AOR = 0.38, 95% CI: 0.22‐0.66), injection safety training (AOR = 0.52, 95% CI: 0.32‐0.84), infection prevention training (AOR = 0.3, 95% CI: 0.18‐0.5), availability of safety box (AOR = 0.04, 95% CI: 0.013‐0.1), Needle recapping (AOR = 0.38, 95% CI: 0.18‐0.81).	Ethiopia	20
Girmaye et al, 2018	Cross sectional	244	Job category (AOR = 0.06, 95% CI = 0.11‐0.28, *P* < 001), educational level (AOR = 33.01, 95% CI = 3.93‐77.07, *P* = .001), work load (AOR = 9.8, 95% CI = 2.68‐35.83, *P* = .001)	Ethiopia	21
Khraisat et al, 2015	Cross sectional	108	Age group (X^2^ = 30.3; *P* < .001) working experience (X^2^ = 20.60; *P* < .001) and marital status (X^2^ = 15.4; *P* = .004).	Jordan	22
Amira, 2014	Cross‐sectional	102	Job category (OR, 2.57 95% CI = 1.39‐4. 76, Work experience (OR 0.30;95% CI = 0.14‐0.63)	Nigeria	23
Archana et al, 2018	Cross‐sectional	950	Workload (*χ* ^2^ = 19 with *P* < .001).	India	24
Chalya et al, 2015	Cross‐sectional	436	Age (OR. 2.65; 95% CI = 1.09‐2.51, *P* < .01), Sex (OR. 3.94; 95% CI = 2.54‐6.83, *P* = .011), Work experience (OR. 3.43; 95% CI = 2.74‐8.56, *P* < .001) and training on safety practices (OR. 4.84; 95% CI = 2.99‐8.72, *P* = .001)	Tanzania	25
Isara et al, 2015	Cross‐sectional	122	Age (OR = 0.28, [CI] =0.11‐0.70)), Work experience (OR = 0.29, CI = 0.11‐0.75)), and being a nurse/job category (OR = 3.38, CI = 1.49‐9.93) or a paramedic (OR = 0.18, CI = 0.06‐0.52)	Nigeria	26
Kebede and Gerensea, 2018	Cross‐sectional	313	Work experience (AOR = 6.321, 95% CI 2.865‐13.948). workload (AOR = 2.903, 95% CI 1.297‐6.498), not use personal protective (AOR = 5.055, 95% CI 2.015‐12.688), did not follow infection prevention guidelines (AOR = 4.623, 95% CI 2.052‐10.416), having infection prevention training (AOR = 5.780, 95% CI 2.691‐12.415)	Ethiopia	27
Khan et al, 2013	Cross‐sectional	497	Work experience (OR = 5.92; 95% CI = 3.45‐10.16, *P* < .001), job category (OR = 2.12; 95% CI = 1.35‐3.32, *P* = .001), working in surgical specialty (OR = 1.6; 95% CI = 1.09‐2.51, *P* < .01)	Pakistan	28
Gabr et al, 2018	Cross‐sectional	2156	Work experience (OR 2.19, 95% CI 1.81 to 2.66), Sex (OR 1.89, 95% CI 1.56 to 2.29), working as a paramedic (OR 1.49, 95% CI 1.03 to 2.25), working in a surgical ward (OR 4.11, 95% CI 1.71 to 9.88), Workload (OR 1.75, 95% CI 1.28 to 2.39), educational sessions (OR 1.99, 95% CI 1.45 to 2.73), absence of policies for NSIs (OR 2.23, 95% CI 1.99 to 2.49), absence of universal precautions (OR 1.66, 95% CI 1.10 to 2.50), needle recapping(OR 2.63, 95% CI 2.12 to 3.26), not using protective clothes (OR 1.39, 95% CI 1.04 to 1.85).	Egypt	29
Weldesamuel et al, 2019	Cross‐sectional	456	Practiced needle recap (AOR = 4.326, 95% CI 2.235, 8.373). Smoking cigarette (AOR = 4.273, 95% CI 1.645, 11.100), work experience (AOR = 4.482, 95% CI 2.189,9.178)	Ethiopia	30

### 3.3 Prevalence of NSI in developing countries

Out of 4235 study participants included in 11 articles provided a quantitative evidence on the prevalence of NSI, 2470 (58.3%) of the healthcare workers were exposed to at least one NSI in their occupational setting. The prevalence of NSI among healthcare workers during the previous 1 year and throughout the career ranged from 19.9% to 54.0%^18^, ^19^, ^21^, ^23^, ^24^, ^25^, ^26^, ^27^, ^30^ and 38.5% to 100%,^18^, ^19^, ^20^, ^23^, ^24^, ^27^, ^28^, ^30^ respectively. Among the studies reported the 1‐year prevalence of NSIs and included 3107 HCWs, 1009(35.7%) were exposed to NSIs^18^, ^19^, ^21^, ^23^, ^24^, ^25^, ^26^, ^27^, ^30^ while the prevalence throughout career exposure among 3433 healthcare workers was 64.1% (2201/3433).^18^, ^19^, ^20^, ^23^, ^24^, ^27^, ^28^, ^30^ In general, the systematic review indicated that more than half of HCWs were exposed to NSI (Table 1).

### 3.4 Factors associated with NSI

The data regarding factors associated with NSI among healthcare workers were extracted from 12 articles and summarized in Table 2 along with their statistical significance (OR, 95% CI, and *P* value).

According to the reviewed articles, statistically significant relationship was found between the prevalence of NSIs and sex,^19^, ^29^ work load,^19^, ^20^, ^21^, ^24^, ^27^, ^29^ working in private hospitals^20^ disassembling of syringe and needle,^20^ needle recapping,^20^, ^29^, ^30^ over use of injection,^20^ universal precaution,^20^, ^27^, ^29^ injection safety training,^20^, ^25^ infection prevention training,^20^, ^25^, ^27^ availability of safety box,^20^ types of professional,^21^, ^24^, ^26^, ^28^ educational level,^21^ age,^22^, ^26^ working experience,^21^, ^23^, ^25^, ^26^, ^27^, ^28^, ^30^ marital status,^22^ personal protective equipment,^27^, ^29^ department/unit,^28^, ^29^ and absence of hospital policies.^29^ See Table 2 below for more details.

## 4 DISCUSSION

Occupational health and safety are vital in every organization, particularly in healthcare settings.^31^ This study reviewed the prevalence of needle‐stick injuries among healthcare workers and factors associated with the prevalence of NSIs in developing countries. In the review, a total of 13 articles that assess the prevalence of NSIs and/or factors associated with NSIs were included.

Out of 4235 study participants included in 11 articles, the majority 2470 (58.3%) of the healthcare workers were exposed to at least one NSI in their occupational setting. This indicates the existence of high risk of exposure to infectious agents such as HBV, HCV, and HIV that can affect the health and wellbeing of HCWs. Depending on the results of various reviewed articles, this systematic review found the prevalence of NSIs ranged from 19.9% to 54.0% with an overall prevalence of 38.5% and 38.5 to 100% with an overall prevalence of 64.1% in the previous 1 year and throughout career time, respectively, that was relatively higher than the finding of another study, reported the overall prevalence of NSIs ranged from 22% to 95%.^32^ Bouya et al, 2020, also reported the global prevalence of NSIs among HCWs that accounts for about 44.5%.^33^ The variation may be due to the difference in sample size and/or methodological quality of included articles or scopes of the studies and/or application of safety standards in their occupational setting, or variation in healthcare system of the countries.

Min Swe et al, 2014 found the overall prevalence of NSI that accounts 19.9%, of which the majority (81%) of the injury was occurred in the medical ward, while Gabr et al, 2018 reported the rate of NSI that accounts for 83.3% of HCWs. Various studies showed a wide variation of NSIs prevalence among healthcare workers in terms of places that was explained by different numbers of healthcare workers in different hospitals, different work cultures, different work environments, and variations in methods of measurement. The variation may be due to the difference in number and occupation of study participants, difference in application of safety precautions or implementation of standards or guidelines, or quality of healthcare system or poor management and environmental conditions of the working areas.

According to CDC, 1987; NIOSH, 2000), the overuse of injections and unnecessary sharp lack of supply, poorly trained staff, recapping needles after use, engineering controls, such as safer needle devices, lack of hazard awareness and training are some of the factors associated with sharp injuries. The overall summary of these reviewed articles also supports this evidence.^18^, ^19^, ^20^, ^21^, ^22^, ^23^, ^24^, ^25^, ^26^, ^27^, ^28^, ^29^

The various studies included in this review also examined various risk factors associated with the prevalence of NSIs. Jahangiri et al (2016) measured associated risk factors based on sex (AOR = 0.24; 95% CI: 0.095‐0.612), work load (OR = 0.86; 95% CI: 0.812‐0.925 while Feleke (2013) measured risk factors based on work load (AOR = 15.58, 95% CI: 7.78 −31.13), working in private hospitals (AOR = 3.03, 95% CI: 1.73‐5.31), disassembling of syringe and needle (AOR = 5.38, 95% CI: 2.68‐10.76), over use of injection (AOR = 5.65, 95% CI: 2.4‐13.3), universal precaution (AOR = 0.38, 95% CI: 0.22‐0.66), injection safety training (AOR = 0.52, 95% CI: 0.32‐0.84), infection prevention training (AOR = 0.3,95% CI: 0.18‐0.5), availability of safety box (AOR = 0.04, 95% CI: 0.013‐0.1), Needle recapping (AOR = 0.38, 95% CI: 0.18‐0.81). Furthermore, Kebede and Gerensea, 2018, considered work experience (AOR = 6.321, 95% CI: 2.865‐13.948), work load (AOR = 2.903, 95% CI: 1.297‐6.498), not use personal protective (AOR = 5.055, 95% CI: 2.015‐12.688), did not follow infection prevention guidelines (AOR = 4.623, 95% CI: 2.052‐10.416) and having infection prevention training (AOR = 5.780, 95% CI: 2.691‐12.415) as a factors for the prevalence of NSIs.

This systematic review revealed that the risk for NSI significantly increased among those who usually or always did needle recapping in comparison to those who did not needle recapping.^29^ Female healthcare workers were at higher risk than males.^19^, ^28^, ^29^ The reviewed articles also reported workload as a potential factor for the prevalence of NSIs.^19^, ^20^, ^21^, ^24^, ^27^, ^29^ Health workers with higher working experience found less likely exposed to NSIs than those with lower working experience,^21^, ^23^, ^25^, ^26^, ^27^, ^28^, ^30^ practice of universal precautions,^20^, ^27^, ^29^ training on infection prevention and safety,^20^, ^25^, ^27^ and working areas/types of profession ^21^, ^24^, ^26^, ^28^ were other factors found as risk factors for the prevalence of NSIs among HCWs of developing countries.

In general, this review found the high prevalence of NSIs and high risk of infectious disease transmission and other complications among HCWs of developing countries. This indicate a need to apply standard precaution, safety practices and other prevention measures developed by OSHAS 18001, CDC, WHO, and others to protect the health of HCWs in their occupational setting. Therefore, applying the following prevention strategies can reduce the occupational exposure to NSI:

1. Establish the objectives and processes in accordance with organization's OH&S policy.
2. Applying and monitoring the implementation of standard precautions guidelines.
3. Creating an appropriate safety and organizational culture among HCWs.
4. Establishing and implementing policies on management of NSIs.
5. Taking continuous corrective actions to improve OH&S performance.

## 5 LIMITATIONS

1. All the included articles were cross‐sectional, and the special methodology limitations of these studies should be considered when interpreting the results.
2. The included studies were carried out in only eight countries of the developing countries.
3. The data were collected in a self‐reported manner in most studies that may have affected the NSI prevalence.
4. Some included articles failed to report either previous 1 year or throughout career time prevalence of NSIs among HCWs.

## 6 CONCLUSION AND RECOMMENDATION

Needle‐stick injuries prevalence and distribution is still a serious public health problem in developing countries. This review indicated that NSIs have been identified as one of the most serious issues that affect the health and well‐being of healthcare workers in the majority of healthcare systems of developing countries.

The majority of reviewed articles reported the risk factors associated with the prevalence of NSIs that include socio‐demographic (age, sex, educational level, etc), behavioral (using personal protective equipment's etc), and institutional factors (availability of facilities). The need for safety practices also stated in almost all reviewed articles. Both local and international government authorities and concerned bodies in collaboration with other private sectors should take action such as training on safety and standard precautions to reduce the risk of NSIs and promote the health of HCWs in different occupational settings of healthcare systems.

## AUTHORS’ CONTRIBUTIONS

DA Mengistu was involved in developing the review idea and designing the review. Both DA Mengistu and ST Tolera performed a literature search. All selected titles and abstracts were independently screened and reviewed by both authors (DA Mengistu and ST Tolera) to confirm its eligibility. DA Mengistu drafted the manuscript while ST Tolera critically revised it. Finally, both authors read and approved the final manuscript.

## DISCLOSURE

Approval of the research protocol: Not applicable. Informed consent: Not applicable. Registry and the Registration No. of the study/Trial: N/A. Animal studies: N/A. Conflict of interest: The authors have no competing interests for this work.

## Data Availability

All data during this review are included in this published article (Tables 1 and 2).
